# Intramolecular Versus Intermolecular Bonding in Drug Gemcitabine and Nucleobases: A Computational Study

**DOI:** 10.3390/molecules30132732

**Published:** 2025-06-25

**Authors:** Natarajan Sathiyamoorthy Venkataramanan, Ambigapathy Suvitha, Ryoji Sahara

**Affiliations:** 1 Department of Chemistry, School of Engineering, Dayanada Sagar University, Bangalore 562 112, India; 2 Department of Chemistry, SKP Engineering College, Tiruvannamalai 606 601, India; suvithaa@gmail.com; 3 Computational Structural Materials Group, Research Center for Structural Materials, National Institute for Materials Science (NIMS), 1-2-1, Sengen, Tsukuba 305-0047, Japan

**Keywords:** drug, nucleobases, non-covalent interactions, DFT calculations, energy decomposition analysis

## Abstract

The adsorption of the drug gemcitabine on nucleobases was investigated using a dispersion-corrected density functional theory (DFT) study. The planar structure of complexes is more stable than those with stacked and buckle-angled configurations. The complexes were found to possess at least two intermolecular hydrogen bonds. The binding energy and interaction energy are both negative, with the highest values observed for the gemcitabine–guanine and the lowest in the gemcitabine–thymine complex. The complex formation was found to be an enthalpy-driven process. Pyrimidine nucleobases have a lower enthalpy of formation than purine nucleobases. The computed HOMA and NICS values on the gemcitabine–nucleobase complexes show a substantial increase compared to the pristine nucleobases. An MESP analysis of the complexes shows a directional interaction and electron density shift between the gemcitabine and the nucleobases. A QTAIM analysis indicates that the intermolecular hydrogen bonds have a partial covalent character. The computed bond energy demonstrates that intermolecular NH⋅⋅⋅N bonds are more potent than other bonds. An energy decomposition analysis using the DLPNO−CCSD(T) method indicates that the complexes exhibit a substantial electrostatic attraction, and dispersion contributes the least towards the system stability. The intermolecular bonds are stronger than the intramolecular bonds in the drug–nucleobase complexes. The strength of intramolecular bonds is determined by the deformation of the gemcitabine ring during the complex formation.

## 1. Introduction

Cancer is the second leading cause of mortality globally, and one of the primary diseases affecting millions of people. Unlike normal cells, cancer cells continue to grow and divide without regulation. Cancer cells spread from the growth site to other parts of the body through the blood or lymphatic system, forming new cancer cells. According to the World Cancer Research Fund International, 510,992 new pancreatic cancer cases were reported in the year 2022. The most commonly used treatments involve chemotherapy, surgery, and radiotherapy, or their combinations [1,2,3]. Chemotherapy remains a fundamental tool because of its simplicity. Among the chemotherapy drugs used, gemcitabine has been an active agent against colon, pancreatic, ovarian, breast, head, neck, and lung cancers [4,5]. Gemcitabine (2′2′-difluorodeoxycytidine, dFdC) is a novel nucleoside analog of deoxycytidine. Originally, gemcitabine (GEM) was produced as an antiviral agent before being established as an anticancer medication based on the remarkable in vitro and in vivo antitumoral activity [6].

Gemcitabine is regarded as a gold standard and the first FDA-approved drug as a monotherapy for pancreatic cancer, which has a long-term survival rate close to zero. The mode of action of gemcitabine arises in the following two pathways: (i) by incorporation into the DNA/RNA inhibiting excision enzyme resulting in replication retardation; (ii) blocks DNA synthesis through inhibition of ribonucleotide reductase enzyme hampering the synthesis of new strands [7]. Gemcitabine is identified as a prodrug, with its activation requiring a series of phosphorylation events. The drug is phosphorylated mainly to gemcitabine monophosphate (dFdCMP) by deoxycytidine kinase, then turning to gemcitabine diphosphate (dFdCDP) and metabolite gemcitabine triphosphate (dFdCTP) by catalysis of other pyrimidine kinases before acting on DNA/RNA [8,9,10]. The half-life of gemcitabine in the human body ranges from 32 to 94 min, during which time it is deaminated to 2′,2′-difluoro-2′-deoxyuridine (dFdU). Cytidine deaminases potentially inactivate gemcitabine by deamination, which can contribute to resistance against the drug in cancer therapy [11,12]. Hence, understanding the mode of interaction of gemcitabine with the nucleobases at the molecular level is essential for improving chemotherapeutic efficacy.

Chemotherapy medications are often classified as either groove binders or DNA intercalators, which can alter the duplex structure’s properties and have therapeutic effects. Historically, computational studies have concentrated on predicting the interaction between DNA nucleobases and pharmaceuticals [13]. Hence, several theoretical studies have been conducted to know the mode and nature of the interaction between 5-fluorouracil and nucleobases [14,15,16,17]. We recently found that 5-fluorouracil bind with Watson–Crick base pairs more firmly than Hoogsteen base pairs due to higher hydrogen bond stability in the former complex [18]. Mazumdar et al. examined the base pairing of two adenine analog nucleobases utilizing various orientations, considering that numerous antiviral medicines are recognized nucleobase analogs [19]. Among the orientations studied, the Watson–Crick base pair possess NH-O/N interaction and contributes more to the interaction energy.

Density functional studies on gemcitabine are limited, and most of them focus on the adsorption and delivery of gemcitabine using nanosystems as drug carriers [20,21,22,23,24]. To understand the mechanisms of the formation of C3′ and C2′ sugar radicals, the one-electron oxidation of gemcitabine has been studied by Adhikary et al. [25]. Theoretical studies have shown that gemcitabine to C3′ conversion needs a substantial barrier, which are in agreement with the experimental studies. Very recently, Malekzadeh et al., using the DFT method B3LYP/6-31+G*, studied the electronic structure of DNA nucleosides, 2′-diflouroro analogues, and gemcitabine [26]. Structural parameters show only a slight variation in the structure, while sugar puckering was noticed in gemcitabine. Ali et al. reported the experimental and computational investigation of gemcitabine with bovine serum albumin (BSA) [27]. Gemcitabine was found to form a 1:1 stable complex with BSA by the MD simulation study. The secondary structure of BSA remained intact while the drug was found to bind at the primary binding site 2.

In the present work, we have carried out a DFT study to understand the binding site of gemcitabine with purine and pyrimidine nucleobases, and the nature of hydrogen bonding that exists between them. To identify the ground state structures, we conducted a thorough search by generating initial structures using the artificial bee colony algorithm. The obtained structures were fully optimized using the M06-2X-D3/6-311+G(d,p) level of theory. Their stability, along with the thermodynamic parameters of their formation, were also computed. The nature of the interaction between the drug and the nucleobases was studied using quantitative molecular electrostatic potential (MESP), aromaticity descriptors, quantum theory of atoms in a molecule (QTAIM), and non-covalent interaction analysis. This work provides a new insight into the intercalation mode of the drug gemcitabine with the nucleobases, and it will be helpful to reveal the mechanism of the drug action, and can further the design of new anticancer drugs.

## 2. Computational Methods

The Gaussian G16 Rev. C.01 software package was used for all density functional theory computations [28]. To create initial geometries, we used the artificial bee colony technique for cluster global optimization (ABCluster) [29,30]. The M06-2X/6-311+G(d,p) approach with Grimme’s D3 dispersion correction was used for the initial optimization [31]. For the thermochemistry of main group elements, the Minnesota functional M06-2X has been tailored to consider dispersion interactions, and performs exceedingly well [32]. Thanthiriwatte et al. showed that meta-GGA functionals M06-2x and functionals with dispersion corrections provide very good potential energy curves for the prototype bimolecular complexes of formic acid, formamide, and foramidine [33]. Furthermore, intermediate and long-range correlation and dispersion must be accounted for when handling hydrogen-bonded systems. To implement implicit solvation effects, the polarizable continuum model (PCM) was used. To ensure that the optimized geometries are at a minimum on the potential surface and to meet the thermodynamic criteria, harmonic vibrational frequencies are calculated for them. At 298 K and 1 atm of pressure, the thermodynamic parameters for the most stable model were computed for both the gas and solution phases. The electronic energy difference between the optimized gemcitabine–nucleobases complex and the sum of the optimized geometries of the gemcitabine molecule and the nucleobases was used to compute the binding energy (BE) of the gemcitabine molecule with the nucleobases.

The wave functions were developed at the M06-2X-D3/6-311+G(d,p) level of theory for topological analysis using the AIMALL program in order to obtain a fuller understanding of the nature of the bonding [34]. The electron density (ρ) and its Laplacian (▽^2^ρ), the total electron energy density (H), the potential electron energy density (V), and the Lagrangian kinetic energy (G) were used to determine the properties of the bond critical point (BCP). Our earlier articles [35] provide more information on the AIM analysis. The Wave Function Analysis–Surface Analysis Suite (WFA-SAS) was utilized to compute and visualize the three-dimensional surface of the quantitative molecular electrostatic potential (MESP) [36,37,38]. Any region’s sign is dictated by the impact of the local electrons (negative) or nuclei (positive). The local greatest positive and most negative values (of which there may be several) of V(r) are denoted as V_S,max_ and V_S,min_, respectively, when plotted on a molecular surface. Using the gauge-independent atomic orbital (GIAO) approach, the ^1^H and ^13^C NMR chemical shift, as well as the nuclear independent chemical shift (NICS) values of stable complexes were calculated in water using the M06-2X/6-311+G(d,p) level of theory [39]. The Multiwfn program was used to perform non-covalent interaction (NCI) and reduced density gradient (RDG) analyses, and the Chemcraft program was used to visualize the results [40,41].

The type of non-covalent interactions that exist between the nucleobases and the gemcitabine molecule was ascertained by means of local energy decomposition (LED) and dispersion interaction density (DID) studies. The optimized shape of B3LYP-D3/6-31G(d,p) was subjected to LED analysis. With the ‘TightPNO’ setting cc-pvdz basis set, we employed the ORCA 6.0 program with the single-point domain-based local pair natural orbital (DLPNO)-based coupled-cluster technique with single, double, and triple excitations (CCSD(T)) computation [42].

## 3. Results and Discussion

### 3.1. Structures and Geometric Parameters of the Gemcitabine–Nucleobase Complexes

**Figure 1:**
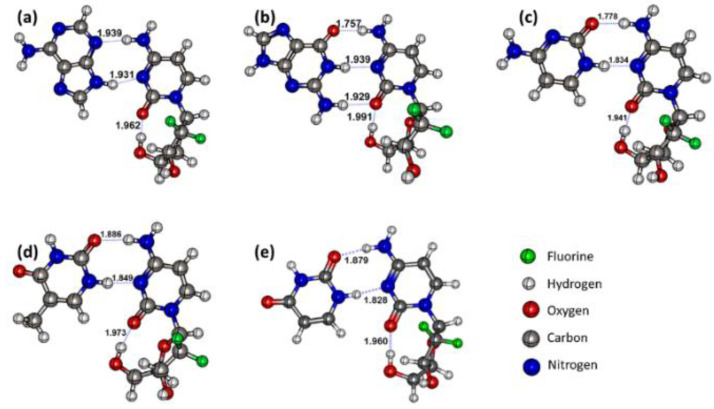
The most stable complexes of gemcitabine with (**a**) adenine, (**b**) guanine, (**c**) cytosine, (**d**) thymine, and (**e**) uracil. Inter- and intramolecular H-bonds are marked with blue lines along with bond length in angstroms.

To know the best mode of interaction of the drug with the nucleobase pairs, the drug gemcitabine was allowed to interact with the nucleobases in all possible modes by taking account of the active electrophilic and nucleophilic sites of the drug gemcitabine and the nucleobases [43,44]. In addition, we employed the artificial bee colony approach for global optimization to ascertain the potential global minimum [45]. Initial conformational sampling revealed ≥16 distinct binding modes per nucleobase. In the Supporting Information in Figures S1–S5, we have provided all the geometries. The most stable geometry of the drug–nucleobase complexes is shown in Figure 1. The purine nucleobase adenine (A) binds with gemcitabine through its N9-H and N3 bonds. The low-lying isomers are shown in the Supporting Information in Figure S1 in their order with decreasing stability. The binding site of gemcitabine was found to vary along with the bond distance to the adenosine. However, all the structures were found to possess at least one intermolecular hydrogen bond. Furthermore, the planar structures have higher stability than those with stacked configurations. In the most stable geometry shown in Figure 1a, the intermolecular hydrogen bonds (HBs) amongst the A and drug molecule were 1.939 and 1.931 Å distance for N3···H-N-g and N9-H···N-g bonding, respectively. It is interesting to notice that the intramolecular hydrogen bond in the gemcitabine drug between the carbonyl group and the hydroxyl group of the sugar moiety remains unchanged during the complex formation, which indicates that neither the carbonyl group nor the sugar moiety is involved in the complex formation.

The drug gemcitabine interacts with the guanine nucleobase in 18 different modes. The most stable mode of interaction exists in planar geometry, and is shown in Figure 1b. In the above geometry, we notice three intermolecular HBs with 1.757, 1.939, and 1.929 Å distances for C6=O6···H-N, N2-H···N-g, and the N1-H···N-g bonding, respectively. The pristine GC base pair C6=O6···H-N distance was longer by 0.017 Å, while the N2-H···N and the N1-H···N distances are shorter by 0.011 and 0.023 Å, which implies the strength of N3···H-N and N9-H···O=C is increased and reduced, respectively [18]. Thus, the introduction of sugar units increases the intermolecular HBs distance. This was further supported by the increased intramolecular HBs distance from 1.962 to 1.991 Å in pristine gemcitabine and the guanine–gemcitabine complex, respectively. The low-lying isomers for the guanine–gemcitabine complex are shown in Figure S2, in which most of the isomers have two or fewer intermolecular HBs or buckle angles. Previous studies of DNA base pairs have shown that planar geometries have higher interaction energy than geometries with buckle angles [46].

The most stable geometries for the pyrimidine nucleobase–gemcitabine complexes are shown in Figure 1c,d. The most stable geometries of the pyrimidine nucleobase–gemcitabine all have two intermolecular HBs and one intramolecular hydrogen bond in the sugar-like moiety of gemcitabine. The intermolecular HB distance in these complexes varies by nucleobase. It is worth pointing out that the thymine and uracil nucleobases differ by a methyl group at the C5 carbon. Furthermore, marginal changes in the intramolecular bond distance are noticed in the gemcitabine drug after complex formation. In the complexes of thymine and uracil with gemcitabine, an elongation in the intermolecular distance by 0.011 and 0.001 Å was observed. By contrast, in the case of the cysteine–gemcitabine complex, the distance was shortened by 0.020 Å. Furthermore, we also notice a change in glycosidic bond angle in all these complexes. Thus, the intermolecular HBs modulate the intramolecular HBs in these complexes.

### 3.2. Energetics

The binding energy and the interaction energy of the gemcitabine drug with the nucleobases are computed at the M06-2X/6-311+G(d,p) level for all studied complexes. The stable geometry, binding energy, and interaction energies are listed in Table 1. The binding energy is derived from the total energy difference between the optimized complex and optimized gemcitabine and the nucleobase. While the interaction energy is calculated as the energy difference between the optimized complex and the single-point energy of gemcitabine and the nucleobase in their complex geometry. Both the binding energy and the interaction energy are negative, indicating that their formation is feasible [47,48].

The other complexes’ relative energies with respect to the most stable geometries are listed in the Supporting Information in Figures S1–S5. Among the complexes, the binding energy is highest for the gemcitabine–guanine complex and least for the gemcitabine–thymine complex. The same trend has been noted in the calculated interaction energy for the 5FU–guanine complexes [49]. The higher binding energy and interaction energy for the gemcitabine–guanine complex indicate that gemcitabine binds with the guanine base more firmly than the other nucleobases. Furthermore, the order of stability was found to match their Gibbs free energy of formation. Incorporation of counterpoise correction tends to increase the binding energy, while the trend remains the same. The basis set superposition error contribution is found to be about 0.009–0.027 kcal mol^−1^ higher in energy. While the percentage of change in contribution was found to change by a maximum of 2.74%, which suggests that the basis set used in the present study would be good enough to study the biomolecules with multiple hydrogen bonds.

A comparison between the binding and interaction energy shows that the interaction energy is low for all complexes except for the gemcitabine–guanine complex. Furthermore, in the solution phase, the binding energy and the interaction energy are lower in the gas phase, likely due to the solvation of the intermolecular HBs, which results in the weakening of the HBs, leading to deformation in the geometry [50]. The computed deformation energy of the nucleobase and the gemcitabine drug during the complexation is provided in Table 1. It lies in the 0.44–1.54 kcal mol^−1^ range, which supports the weakening of the HBs during solvation.

The deformation energy is generally higher for the gemcitabine drug than for the nucleobases, probably due to the presence of flexible sugar units. Among the complexes, the deformation energy is higher for the gemcitabine–guanine complex. The thermodynamic quantities for the interaction among gemcitabine and the nucleobases were calculated, and are shown in Table 1. It is apparent from the table that the thermodynamic parameters, such as enthalpy, were negative for all the studied complexes, while the Gibbs free energy is negative for all the complexes except the gemcitabine–adenine complex. The negative values for enthalpy and Gibbs free energy indicate that gemcitabine complexation with the nucleobase is exothermic and spontaneous. Furthermore, the change in enthalpy and Gibbs free energy of formation of the complexes are higher for the gemcitabine–guanine complex. The enthalpy of formation for the pyrimidine complexes is lower than that of the purine complexes. In general, the change in enthalpy is higher for all complexes, indicating that the complex formation is an enthalpy-driven process. Thus, the gemcitabine drug complexation with the nucleobases is more facile and spontaneous, except for the gemcitabine–adenine complex, which is an enthalpy-driven process. Earlier, Rezaei-Sameti and Borojeni reported that the cytosine and guanine bases pair has the highest enthalpy for the formation of the 5-Fluorouracil–nucleobase complex [14]. In the present study, gemcitabine shows higher selectivity towards the guanine base, as it is a cytidine analogue.

### 3.3. Frontier Molecular Orbitals and Conceptual DFT

The two most important orbitals that promote a chemical reaction are the lowest unoccupied molecular orbital (LUMO) and the highest occupied molecular orbital (HOMO). The ability to contribute an electron is represented by the HOMO orbital, whereas the ability to receive the electron is determined by the LUMO [51,52,53]. Consequently, the electrical stability of the system can be predicted using the frontier molecular orbital (FMO) gap, specifically the HOMO–LUMO gap, in a compound. Additionally, Koopmans’ theorem can be used to estimate the quantum chemical descriptors, such as chemical hardness (η), chemical potential (μ), electrophilicity index (ω), and softness (σ). The following Equations (1)–(5) can be used to calculate the aforementioned quantum chemical descriptors:

η = IP − EA ≈ E_LUMO_ − E_HOMO_ (1)

μ = −χ = (IP + EA)/2 ≈ (E_HOMO_ + E_LUMO_)/2 (2)

ω = μ^2^/2η ≈ (E_HOMO_ + E_LUMO_)2/(4 (E_LUMO_ − E_HOMO_)) (3)

σ = 1/(IP − EA) = 1/η ≈ 1/(E_LUMO_ − E_HOMO_) (4)

ΔN = −μ/η(5)

The computed chemical descriptors for the gemcitabine–nucleobase complexes are provided in Table 2. The energy gap for the nucleobases was in the range of 6.92–8.12 eV, while gemcitabine has an E_gap_ of 7.82 eV. The drug complex lies in the range of 5.96–7.15 eV, which is less than the pristine nucleobase and the drug gemcitabine, which reveals that drug complexes are more chemically reactive towards further reaction with another nucleobase or the gemcitabine drug, which is less likely. The chemical hardness values are more prominent for the complexes than the nucleobases, and follow the trend gemcitabine–uracil > gemcitabine–thymine > gemcitabine–cytosine > gemcitabine–adenine > gemcitabine–adenine. Consequently, the gemcitabine–uracil complexes are less likely to undergo electron transfer, and are less sensitive than the nucleobases. Also, the term softness supports the prior conclusion on reactivity.

The electrophilicity index (ω) is a positive measure, and high values are features of most electrophilic systems [54,55] The electrophilicity value of adenine is the lowest among the nucleobases studied. The gemcitabine–thymine and gemcitabine–uracil complexes have the highest values in the drug–nucleobase complexes. Thus, one can anticipate that the above complexes are more electrophilic than the other drug–nucleobase complexes. The chemical potential (μ) in a system designates the escaping tendency of an electron cloud [56]. When complex formation occurs, the electrons flow from the higher chemical potential to the lower species until the electronic chemical potential becomes equal. The free drug gemcitabine has a chemical potential of 4.55 eV, while the nucleobases have chemical potential values in the range of 3.78–4.64 eV.

Compared to the pristine nucleobase and the gemcitabine drugs, the drug–nucleobase complexes have lower chemical potential, making the latter less proficient at attracting electrons. We have calculated the charge transfer based on electrophilicity using Equation (6) to ascertain the direction of charge transfer.

ECT = (ΔN_gemcitabine_) − (ΔN_nucleobase_) (6)

In the case of ECT < 0, gemcitabine acts as an electron donor; if ECT > 0, then gemcitabine acts as an electron acceptor. For the gemcitabine–nucleobase complexes, all values are negative, indicating that gemcitabine gains electrons during complex formation. The above results are further proved by the existence of the HOMO and LUMO orbitals observed on the complex. The HOMO and LUMO orbitals of the gemcitabine–nucleobase complexes and the pristine nucleobase and gemcitabine are shown in Figure S6. It can be seen that, in all the studied complexes, the HOMO is concentrated on the surface of the nucleobases, and the LUMO is localized on the surface of the gemcitabine drug. Thus, one can anticipate that the nucleobase of the gemcitabine–nucleobase complex can undergo an electrophilic attack, and the gemcitabine drug can undergo a nucleophilic attack when subjected to further chemical reactions.

### 3.4. Nature of Interactions

The interactions between the molecules help one predict and manipulate the behavior of the substances at the molecular level. To understand the nature of the intermolecular interactions between the drug and the nucleobases, we performed a quantitative molecular electrostatic potential (MESP) analysis [57], an atoms-in-molecules (AIM) analysis [58], and a non-covalent interaction–reduced density gradient (NCI–RDG) analysis [59]. Hydrogen bonding interactions have been extensively demonstrated to enhance cyclic 4*n* + 2π electron delocalization and augment aromaticity [60]. Therefore, we computed the harmonic oscillator model of aromaticity (HOMA) and the nucleus independent chemical shifts (NICS) analyses for the gemcitabine–nucleobases and compared them with the drug–nucleobase complexes to comprehend the hydrogen bonding between the drug gemcitabine and the nucleobases.

#### 3.4.1. π-Electron Delocalization Indicators and Molecular Electrostatic Potential

It is well known that hydrogen bonds in DNA base pairs are strongly affected by π-electron delocalization (π-ED), which is not directly measurable but can be evaluated with other molecular properties, including energetic, structural, and magnetic properties [61]. In the present study, we have calculated the harmonic oscillator model of aromaticity (HOMA) and the nucleus independent chemical shifts (NICS) analyses for the nucleobases and the drug gemcitabine and related them with the gemcitabine–nucleobase complexes.

The computed HOMA, NICS(0), and NICS(1) values are provided in Table 3. In general, the HOMA value of gemcitabine was found to increase upon complexation with the nucleobases. In the gemcitabine–nucleobase complex, the HOMA readings of both 6-membered and 5-membered rings were observed to rise. However, the highest increase was observed on the 6-membered ring of the gemcitabine–guanine complex. In the pyrimidine base complex, the cytosine ring showed a significant increase in the HOMA value after complexation with cytosine, which is determined by the observed binding energy values of the system. Previous studies have reported that binding energies of complexes are proposed to increase the HOMA values [62].

The NICS is an essential parameter for assessing the π-ED of aromatic and pseudo-aromatic molecules. The magnitude of electron delocalization is directly related to the total value of the NICS(1). It is well documented that, when the NICS(1) value of a molecule increases, the π-ED increases [16]. The computed NICS(1) values on the gemcitabine–nucleobase complexes show a substantial increase compared to the pristine nucleobases. The increase in the NICS(1) values was highest for the gemcitabine–guanine and gemcitabine–cytosine complexes, which are by the binding energy and the HOMA values.

**Figure 2:**
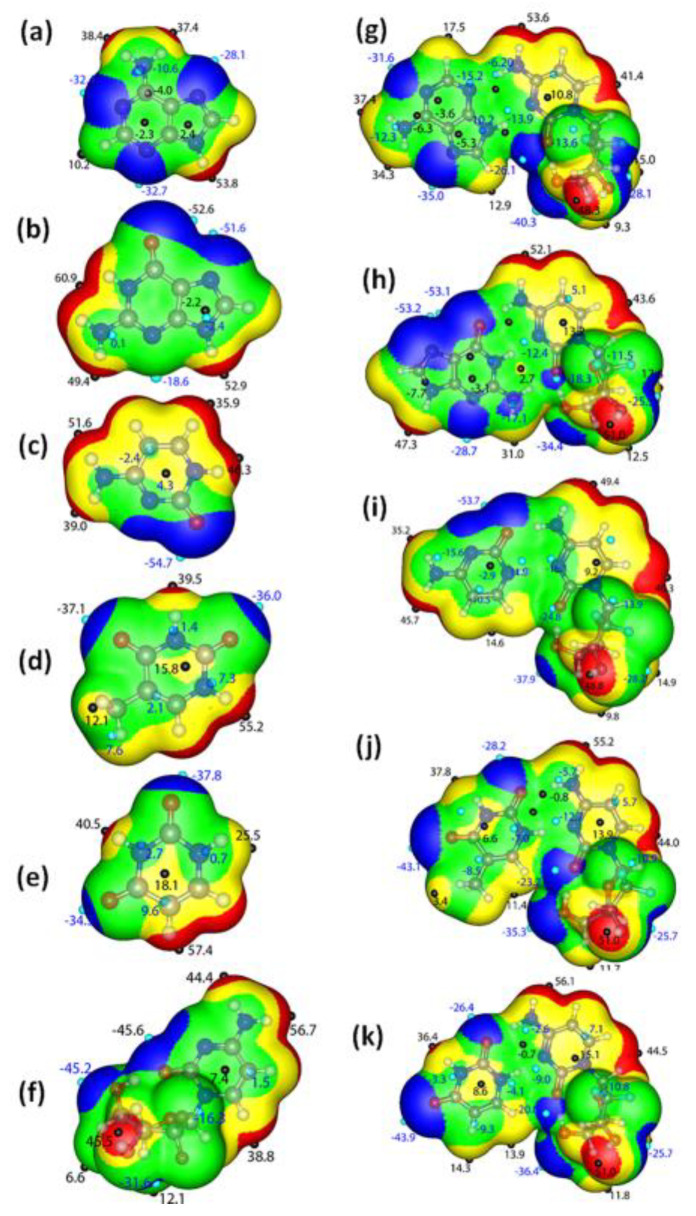
Molecular electrostatic surface potentials mapped on the corresponding 0.001 a.u. electron density isosurface: (**a**) A, (**b**) G, (**c**) C, (**d**) T, (**e**) U, (**f**) gemcitabine, (**g**) gemcitabine–A, (**h**) gemcitabine–G, (**i**) gemcitabine–C, (**j**) gemcitabine–T and (**k**) gemcitabine–U complexes.

The molecular electrostatic potential (MESP) helps us to understand the attraction between individual molecules, as charge transfer/electrostatic interactions are known to be one of the significant driving forces during complex formation [63,64]. In Figure 2, the quantitative electrostatic potentials for the bases, gemcitabine, and gemcitabine–nucleobase complexes are provided and mapped on a 0.001 electrons/Bohr^3^ isodensity surface. In the above figures, the red color represents the electrophilic area, while the blue and green color depicts nucleophilic and neutral regions in the molecules. These electrophilic and nucleophilic regions are designated as V_s,max_ and V_s,min_ [65]. In the nucleobases and the gemcitabine drug, the V_s,max_ is observed on the hydrogen atom of the pyrrole-like ring and the hydrogen atoms of the amino group. As gemcitabine resembles that of cytosine, the MESP regions of gemcitabine are similar to those of cytosine; however, the V_s,max_, and V_s,min_ values differ a little due to the presence of an extra sugar moiety in gemcitabine. In all the nucleobases and the gemcitabine drugs, the V_s,max_ are observed on the imine nitrogen and the oxygen atom of the carbonyl group.

It is commonly known that the system becomes unstable when sections of the same potential are brought together [66]. A directional interaction with opposite potentials leads to the establishment of the most stable complex. Owing to the directional interaction, the positive potential regions will interact with the negative region, leading to a decrease/increase in positive/negative potential values. The MESP of the gemcitabine–nucleobase complexes are visualized in Figure 2g–k. In the gemcitabine–adenine complex, the most positive value observed on the pyrrole-like hydrogen atom of adenine matches with the most negative potential value observed on the imide nitrogen atom of the gemcitabine drug. Furthermore, in the complex, the V_s,min_ value observed on the carbonyl oxygen was reduced to −26.1 from −45.6 kcal mol^−1^ in pristine gemcitabine. This indicates the carbonyl group’s participation in complex formation, leading to an electron density shift. A similar supposition can be arrived at while observing the MESP potential values of all other stable complexes. Thus, during complex formation, a directional interaction along with an electron density shift occurs to help form a stable complex.

#### 3.4.2. Atom in Molecule Analysis and Non-Covalent Interaction Analysis

**Figure 3:**
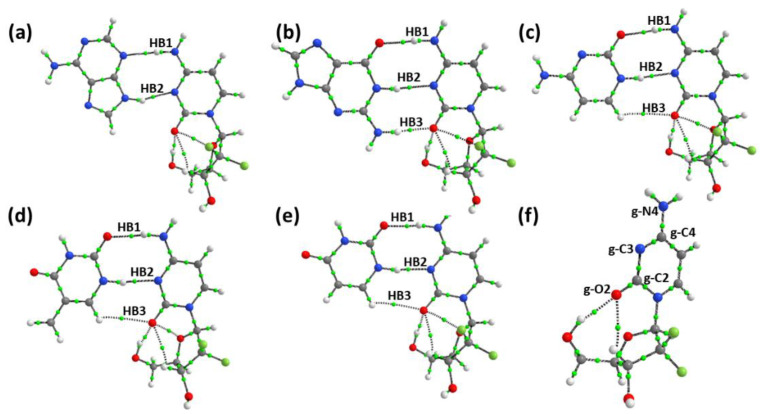
Molecular topography analysis for (**a**) gemcitabine–adenine, (**b**) gemcitabine–guanine, (**c**) gemcitabine–cytosine, (**d**) gemcitabine–thymine, (**e**) gemcitabine–uracil, and (**f**) gemcitabine.

The quantum theory of atoms in molecules (QTAIM) is used to examine the nature of interactions in many molecular systems and to categorize and understand bonding interactions [67]. The QTAIM is also used to compute the individual hydrogen bond strength if the topological parameter potential energy at the bond critical point (BCP) is known [68]. The various topological parameters at the BCPs, namely electron density (ρ(r)), its Laplacian (∇^2^ρ(r)), and the total electron density (H(r)), allow us to classify the interactions. For hydrogen bonds, the Laplacian (∇^2^ρ(r)) is positive, the potential energy (V(r)) is negative, and the kinetic energy density G(r) and H(r) are positive values. If a bond has positive ∇^2^ρ(r) and H(r), a weak noncovalent hydrogen bond is proposed. The tomographs obtained for all of the stable gemcitabine–nucleobase complexes are shown in Figure 3.

It can be observed that the total number of intramolecular BCPs on gemcitabine increases from two to three in both pristine gemcitabine and the gemcitabine–nucleobase complexes. During the complexation, an additional intramolecular BCP between the fluorine and oxygen atoms arises due to the structural deformation of gemcitabine during complexation. It is worth pointing out that the computed deformation energies are higher on gemcitabine than on the nucleobase.

The topological values for the intermolecular interactions between gemcitabine and the nucleobases are provided in Table 4. The ρ(r) values for all the intermolecular BCPs were in the range of 0.004–0.037 a.u., which is close to the value proposed for hydrogen bonding. The ρ(r) value is highest for the N-H⋅⋅⋅O=C type of interaction and lowest for the C=O⋅⋅⋅H-C interaction. The ∇^2^ρ(r) values are positive for all the intermolecular BCPs, while the H(r) values are negative for most bonds. The negative H(r) values propose the supremacy of potential energy over kinetic energy. H(r) with negative values for intermolecular BCPs shows a partial covalent character.

The gemcitabine molecule shows intramolecular BCPs, with the oxygen atom of the carbonyl in pyrimidine, bonding with the hydroxyl, alkyl, and fluoro groups. The intramolecular BCPs have ρ(r) values of 0.009–0.023 a.u. Among the intramolecular bonds in gemcitabine, the hydroxyl–carbonyl oxygen interaction has the highest ρ(r) values and remains significantly unchanged after complexation with the nucleobases. The ρ(r) values on the alkyl hydrogen–carbonyl oxygen in complexes are lower compared to the free gemcitabine molecule. The intramolecular F⋅⋅⋅O bond has ρ(r) values in the range of 0.009–0.014 a.u., which is closer to the value reported for the Cl⋅⋅⋅O intramolecular bond. It is interesting to see that the ρ(r) values of F⋅⋅⋅O are higher for the complexes with larger binding energy, the gemcitabine–guanine complex, while the other intramolecular bonds remain nearly unchanged. The existence of weak interactions between electronegative atoms is not uncommon. A comparison between the inter- and intramolecular ρ(r) values shows that the latter are weak. The higher deformation energy of gemcitabine and higher ρ(r) values of F⋅⋅⋅O in the gemcitabine–guanine complex suggest that the alkyl chain plays a significant role in the complex formation process.

**Figure 4:**
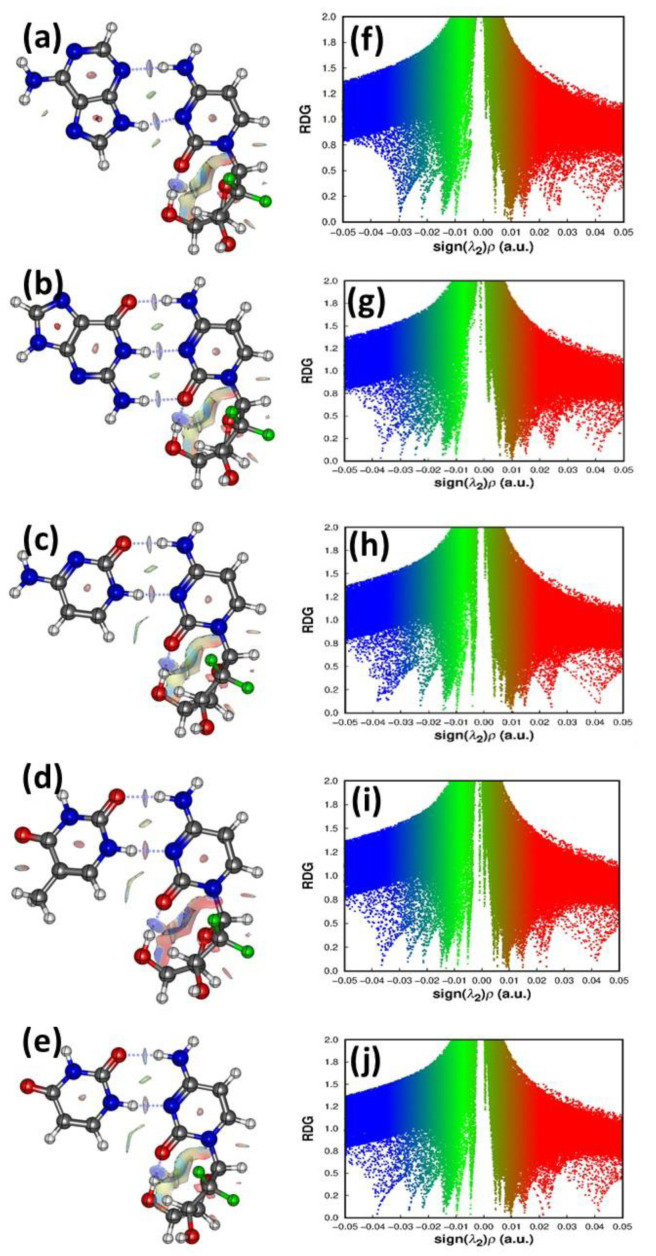
NCI plots of (**a**) gemcitabine-adenine (**b**) gemcitabine-guanine (**c**) gemcitabine-cytosine (**d**) gemcitabine-thymine and (**e**) gemcitabine-uracil complex and RDG of (**f**) gemcitabine-adenine (**g**) gemcitabine-guanine (**h**) gemcitabine-cytosine (**i**) gemcitabine-thymine and (**j**) gemcitabine-uracil complex depicting non-covalent interaction for the most stable complexes.

The ratio of |G/V| value helps one to decide the electrostatic or covalent nature of the HB. The computed values for the intermolecular HBs lie in the range of 0.90–1.26. These values confirm that the bonds have a partially covalent character. The ellipticity at the BCP is used as a measure of bond stability as it provides the extent of charge accumulation at the BCP. A BCP with a higher ellipticity suggests that the bond can undergo rupture. Hence, electrostatic interactions with the van der Waals interactions have higher ellipticity than hydrogen bonds [69]. From Table 4, it is evident that the C=O⋅⋅⋅H-C6 bonds have higher ellipticity values and hence are weaker in strength than the N3⋅⋅⋅H-N and the NH⋅⋅⋅N3 bonds. Computing the individual hydrogen bond strength is intimidating; however, using the AIM analysis and the Espinosa–Molins–Lecomte (EML) semi-empirical equation, we computed the hydrogen bond energy (E_HB_), which is approximately half the value of the potential energy density at the BCPs. It should be pointed out that potential energy density describes the attractive component of the total energy density at the BCP. The computed intermolecular E_HB_ values in kcal mol^−1^ are provided in Table 4. The NH⋅⋅⋅N bond strength ranges from 6.87 to 10.64 kcal mol^−1^. An NCI analysis is a significant method for identifying non-covalent interactions within a molecule [70]. It involves hydrogen bonding, steric effects, and the presence of van der Waals forces. The attractive, repulsive, and van der Waals inter- and intramolecular interactions are deciphered from the sign of λ_2_. In Figure 4a–e, the RDG isosurface for the stable drug nucleobases is provided. The red regions depict the repulsive interactions. The green and brown regions portray the attractive van der Waals interaction, with the green region being more substantial than the brown one. The blue color patches confirm the strong hydrogen bond interactions. The presence of brown regions is due to the rings possessing π-electrons. In the pyrimidine bases of the drug, the presence of additional H-bonding is confirmed by the green and blue isosurfaces between the H and carbonyl O atoms of the gemcitabine drug. The occurrence of several regions with red, green, and brown colors implies the coexistence of repulsive and attractive interactions, which can lead to anti-cooperative effects [71].

Figure 4f–j, depicts the plots of the RDG vs. the electron density multiplied by the sign of λ_2_ for the stable drug–nucleobase complexes. For the pristine nucleobases and the gemcitabine drug, the same is shown in the Supporting Information Figure S7. The spike regions corresponding to the steric effect exhibit no noticeable change in the pattern, confirming that there is no apparent change in the steric effect after the adsorption of gemcitabine to the nucleobase. However, in the spike regions involving the H-bonding interactions (−0.010–−0.025 a.u.), many new spikes were produced along with an increase in their strength. This supports that the H-bonding interactions are stronger and play a dominant role in stabilizing the complexes [72].

#### 3.4.3. Dispersion Interaction Density (DID) and Local Energy Decomposition (LED) Analyses

**Figure 5:**
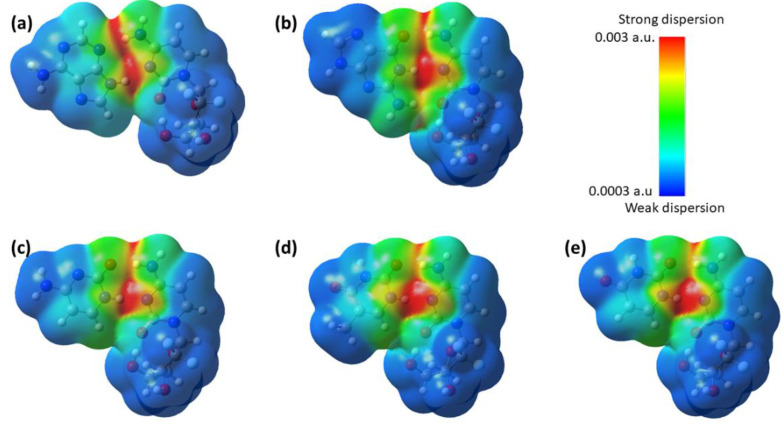
Dispersion interaction density profile for the complexes (**a**) gemcitabine–adenine, (**b**) gemcitabine–guanine, (**c**) gemcitabine–cytosine, (**d**) gemcitabine–thymine, and (**e**) gemcitabine–uracil.

A dispersion interaction density (DID) analysis helps for visualizing weak van der Waals (vdW) interactions in complexes [73]. The DID values are projected onto an isodensity surface, obtained from the total molecular density, and are used as a simple technique for visualizing the most critical contacts that contribute to the van der Waals (vdW) interaction of any complex. The DID profile obtained for the complexes is shown in Figure 5, where the red zones indicate strong dispersion interactions and the blue zones indicate weaker dispersion interactions.

The DID profile revealed that attractive dispersion interactions occur along the interface between gemcitabine and the nucleobases, with the strongest interactions observed between the hydrogen atoms of the amine group of gemcitabine and the nitrogen and carbonyl oxygen atoms of the nucleobases. In addition, the imide group of the nucleobases has the strongest interaction with the nitrogen atom of gemcitabine. Furthermore, the presence of green zones indicates weak attractive dispersion interactions between the alcohol group of gemcitabine and the C-H/N-H group of the nucleobases. A comparison between the purine and pyrimidine nucleobases reveals that the red zones are more concentrated in the purine bases; whereas, in the pyrimidine bases, they are more dispersed.

In order to gain a quantitative measurement on the nature of interactions in these complexes, we carried out local energy decomposition (LED) calculations. The LED analysis provides a breakdown of the domain-based local pair natural orbital CCSD(T) [DLPNO–CCSD(T)] energy into additive contributions representing the interaction between pairs of user-defined fragments [74,75]. In LED, the total binding energy between the fragments can be written as in Equation (7):

ΔE = ΔE_int_ + ΔE_geo-prep_ (7)

where ΔE_int_ is the interaction energy between the fragments considered, and ΔE_geo-prep_ is the deformation energy.

The ΔE_int_ can be decomposed into Hartree–Fock ($\Delta E_{int}^{HF}$) and correlation contributions ($\Delta E_{int}^{C}$) as follows:

(8) $$\Delta E_{\mathrm{int}}=\Delta E_{int}^{HF}+\Delta E_{int}^{C}$$

The Hartree–Fock and correlation contributions can be further disintegrated as follows:

(9) $$\Delta E_{int}^{HF}=\Delta E_{el-prep}^{HF}+\Delta E_{elstat}^{frags}+\Delta E_{exch}^{frags}$$

(10) $$\Delta E_{int}^{C}=\Delta E_{non-dispersion}^{C-CCSD}+\Delta E_{dis}^{C-CCSD}\;+\Delta E_{int}^{C-\left(T\right)}$$

In Equation (9), $\Delta E_{el-prep}^{HF}$ is the Hartree–Fock contribution to the electronic preparation of each fragment in the complex, and is always a positive term. The terms $\Delta E_{elstat}^{frags}$ + $\Delta E_{exch}^{frags}$ define the electrostatic and exchange interactions, while $\Delta E_{non-dispersion}^{C-CCSD}\;$ is a summation term of weak pair and strong pair dispersion interactions between fragments, and $\Delta E_{int}^{C-\left(T\right)}$ is the triples correction to the interaction energy, which includes the electronic preparation and interfragment interaction energies.

The DLPNO–CCSD(T)–LED calculations for the gemcitabine–nucleobase complexes were performed using the cc-pVDZ basis set on the B3LYP–D3/6–31G(d,p) optimized geometry. The results obtained in the LED calculations are presented in Table 5. The ΔE_geo-prep_ values for the complexes follow the same trend as the computed summation deformation energy of the nucleobase and gemcitabine [76]. The ΔE_geo-prep_ values are more significant for the gemcitabine–guanine complex, and least for the gemcitabine–adenine complex. Expectedly, the ΔE_geo-prep_ values are far smaller than the ΔE_int_ values. Furthermore, the ΔE_int_, which is interaction energies computed at the HF level, is far more significant than the correlation interaction contribution obtained for the complexes. It is evident from Table 5 that the sum of the attractive electrostatic preparation energy $\Delta E_{elstat}^{ref}$ and the exchange energy $\Delta E_{exch}^{ref}$ is almost fully compensated by its repulsive electronic preparation term $\Delta E_{el-prep}^{ref}$. Dispersion contributes the least to interaction energy, and electrostatic contributes the most. Furthermore, the larger electrostatic term $\Delta E_{elstat}^{ref}$ of the gemcitabine–guanine complex, accounts for its higher stability among the complexes.

### 3.5. Role of Intramolecular Hydrogen Bond of Gemcitabine in Stabilizing the Complex

To understand the role of the flexible sugar moiety of gemcitabine in the complex formation, the intramolecular interactions that exist between the hydroxyl group/alkyl hydrogen/fluorine and the carbonyl oxygen were studied using NBO analysis and QTAIM theory. The selected interactions are listed in Table 6. The gemcitabine molecule is stabilized by the transfer of a lone pair of electrons located on the oxygen atom of the carbonyl group to the σ orbital of the hydroxyl group with a stabilization energy of 3.03 kcal mol^−1^. The computed EML-based hydrogen bond energy for the interaction was 5.46 kcal mol^−1^. In addition, the carbonyl oxygen atom donates electrons to the σ orbital of the alkyl hydrogen atom, stabilizing by 0.53 kcal mol^−1^, while its E_HB_ was 3.08 kcal mol^−1^. Furthermore, the computed E_HB_ values are nearly equal in strength to that of a CH⋅⋅⋅O bond, and the combined values are found to be nearly equivalent to the conventional intramolecular hydrogen bond observed between the –OH and the carbonyl oxygen atom. Thus, charge transfer alone does not act as a predominant factor in deciding the strength of the intramolecular bonds. The fixable sugar moiety undergoes a more significant structural deformation, and this cooperative effect improves the intramolecular bond strength.

## 4. Conclusions

In summary, the interaction between the drug gemcitabine and the nucleobases has been explored by means of the dispersion-corrected density functional theory study. The binding site of gemcitabine was found to vary along with the nucleobase, and all the most stable structures were found to possess at least two intermolecular hydrogen bonds. In the pyrimidine nucleobase–gemcitabine complexes, a change in glycosidic bond angle was observed. The binding energy and the interaction energy are all negative and are maximum for the gemcitabine–guanine complex, and minimum for the gemcitabine–thymine complex. Due to the presence of flexible sugar units, the deformation energy is higher for the gemcitabine drug than for the nucleobases. The enthalpy for the formation of all the complexes was negative, while the Gibbs free energy was positive for the gemcitabine–adenine complex. The complex formation was found to be an enthalpy-driven process. Pyrimidine nucleobases have a lower enthalpy of formation than purine nucleobases. The order of stability was found to match their Gibbs free energy of formation.

The nature of the interaction between the drug gemcitabine and the nucleobases was identified using the MESP, AIM, HOMA, NICS, and NCI–RDG analyses. In the gemcitabine–nucleobase complex, the HOMA values of 6- and 5-membered rings increased compared to the pristine nucleobase. The highest increase was observed on the 6-membered ring of the gemcitabine–guanine complex. The computed NICS(1) values on the gemcitabine–nucleobase complexes showed a substantial increase compared to the pristine nucleobases. In the gemcitabine–nucleobase complex, the most positive value was observed on the pyrrole-like hydrogen atom of the nucleobase matches, with the most negative potential value observed on the imide nitrogen atom of the gemcitabine drug. Furthermore, in the complex, the V_s,min_ value observed on the carbonyl oxygen was reduced substantially compared to pristine gemcitabine. Thus, a directional interaction and electron density shift occurred during the complex formation to help form a stable complex. The QTAIM analysis showed the presence of two/three intramolecular BCPs in the gemcitabine–nucleobase complexes, and an additional intramolecular BCP between the fluorine and the oxygen atoms arose due to the structural deformation of gemcitabine during complexation. The intermolecular hydrogen bonds showed a partial covalent character. The computed bond energy showed that the intermolecular NH⋅⋅⋅N bond is stronger than the other bonds. Among the intramolecular bonds, the electrostatic interaction between F and carbonyl oxygen was the weakest bond observed. The above results are supported by the NCI–RDG analysis. The intermolecular bond strength between the gemcitabine–nucleobase complexes is about two-times higher in energy than the intramolecular bond strength observed in the gemcitabine drug. Though the present work cannot be used to understand the drug interactions with DNA and enzymes, this work provides a new insight into the intercalation mode of the drug gemcitabine with the nucleobases, and it will be helpful to reveal the mechanism of drug action and the further design of new anticancer drugs.

## Figures and Tables

**Table 1:** Computed binding energy, interaction energy, deformation energy, and thermodynamic parameters for the formation of the gemcitabine–nucleobase complexes for stable complexes.

Nucleobase	Binding Energy * (kcal mol^−1^)		Interaction Energy (kcal mol^−1^)		Deformation Energy (kcal mol^−1^)		ΔH (kcal mol^−1^)	ΔG (kcal mol^−1^)
	Gas	Solution	Gas	Solution	Nucleobase	Gemcitabine		
Adenine	−18.83 (−19.05)	−10.79	−21.33	−5.28	0.44	0.91	−10.37	0.33
Guanine	−26.51 (−27.26)	−14.84	−31.25	−17.49	1.18	1.54	−14.39	−3.14
Cytosine	−21.82 (−22.25)	−11.69	−25.53	−13.44	1.04	1.19	−11.27	−0.61
Thymine	−19.93 (−20.15)	−11.56	−23.39	−13.27	0.94	1.14	−11.14	−0.34
Uracil	−20.68 (−20.88)	−11.63	−23.66	−13.35	0.84	0.89	−11.21	−0.55

* Values in the parentheses are BSSE corrected values.

**Table 2:** The calculated band gap (E_gap_ (in eV)) and the reactive descriptors (in eV) for gemcitabine, the nucleobase, and the gemcitabine–nucleobase complexes at the M06-2X-D3/6-311+G(d,p) level of theory.

System	E_gap_	μ	η	ω	σ	ΔN
Gemcitabine	7.827	4.558	7.827	1.327	0.128	−0.582
Adenine	7.670	3.787	7.670	0.935	0.130	−0.494
Guanine	6.929	3.791	6.929	1.037	0.144	−0.547
Cytosine	7.772	4.150	7.772	1.108	0.129	−0.534
Thymine	7.862	4.394	7.862	1.228	0.127	−0.559
Uracil	8.129	4.641	8.129	1.325	0.123	−0.571
Gemcitabine–Adenine	6.658	4.132	6.658	1.282	0.150	−0.621
Gemcitabine–Guanine	5.959	3.888	5.959	1.268	0.168	−0.652
Gemcitabine–Cytosine	7.025	4.234	7.025	1.276	0.142	−0.603
Gemcitabine–Thymine	6.845	4.390	6.845	1.408	0.146	−0.641
Gemcitabine–Uracil	7.150	4.579	7.150	1.466	0.140	−0.640

**Table 3:** Computed HOMA and NICS aromaticity indices for gemcitabine, the nucleobases, and the most stable gemcitabine–nucleobase complexes.

Complex	Gemcitabine			A/G			C/T/U		
	HOMA	NICS(0)	NICS(1)	HOMA (6/5)	NICS(0) (6/5)	NICS(1) (6/5) *	HOMA	NICS(0)	NICS(1)
Gemcitabine	0.676	−1.488	−3.668	-	-	-	-	-	-
Adenine	-	-	-	0.981/0.881	−6.304/−11.455	−8.946/−10.199	-	-	-
Guanine	-	-	-	0.700/0.883	−2.628/−11.481	−3.719/−9.721	-	-	-
Cytosine	-	-	-	-	-	-	0.672	−0.541	−3.010
Thymine	-	-	-	-	-	-	0.466	−1.079	−2.226
Uracil	-	-	-	-	-	-	0.499	−0.661	−1.814
Gemcitabine–Adenine	0.693	−1.376	−3.254	0.983/0.912	−6.346/−11.727	−8.703/−10.675	-	-	-
Gemcitabine–Guanine	0.703	−1.568	−3.396	0.824/0.875	−3.166/−11.001	−4.141/−9.385	-	-	-
Gemcitabine–Cytosine	0.693	−1.549	−3.377	-	-	-	0.794	−1.441	−3.833
Gemcitabine–Thymine	0.701	−1.616	−3.372	-	-	-	0.540	−1.315	−2.422
Gemcitabine–Uracil	0.706	−1.658	−3.480	-	-	-	0.574	−0.951	−2.187

* represents values on the 6- and 5-membered rings.

**Table 4:** Topological parameters of intermolecular bonds obtained from the AIM analysis for the stable gemcitabine–nucleobase complexes. The values of topological parameters are provided in a.u. and bond length in Å; The value of E_HB_ is provided kcal/mol.

BCP	Bond Length	ρ	∇^2^ρ	λ_1_	λ_2_	λ_3_	V	G	H	E_HB_	Ellipticity
Gemcitabine–Adenine complex											
HB1	1.939	0.02981	0.08956	−0.04274	−0.04004	0.17233	−0.02203	0.02221	−0.00018	6.91	0.07
HB2	1.931	0.02988	0.09520	−0.04237	−0.04013	0.17770	−0.02290	0.02335	−0.00045	7.18	0.06
Gemcitabine–Guanine complex											
HB1	1.757	0.03704	0.13148	−0.05969	−0.05781	0.24897	−0.03392	0.03340	0.00053	10.64	0.03
HB2	1.939	0.02992	0.09284	−0.04263	−0.03997	0.17543	−0.02244	0.02282	−0.00039	7.04	0.07
HB3	1.929	0.02475	0.09970	−0.03412	−0.03222	0.16603	−0.01977	0.02235	−0.00258	6.20	0.06
Gemcitabine–Cytosine complex											
HB1	1.778	0.03512	0.12700	−0.05529	−0.05339	0.23568	−0.03151	0.03163	−0.00012	9.88	0.04
HB2	1.834	0.03815	0.10525	−0.06008	−0.05632	0.22164	−0.03214	0.02922	0.00291	10.08	0.07
Gemcitabine–Thymine complex											
HB1	1.886	0.02698	0.10566	−0.03861	−0.03682	0.18109	−0.02193	0.02417	−0.00224	6.87	0.05
HB2	1.849	0.03667	0.10455	−0.05674	−0.05278	0.21407	−0.03045	0.02829	0.00216	9.55	0.08
Gemcitabine–Uracil complex											
HB1	1.879	0.02719	0.10691	−0.03916	−0.03753	0.18360	−0.02224	0.02448	−0.00225	6.98	0.04
HB2	1.828	0.03849	0.10526	−0.06085	−0.05704	0.22315	−0.03259	0.02945	0.00314	10.23	0.07

**Table 5:** DLPNO–CCSD(T) based local energy decomposition of the binding energy of gemcitabine on the nucleobases into various energy components (kcal mol^−1^).

System	ΔE_int_	ΔE_geo-prep_	$\Delta E_{el-prep}^{ref}$	$\Delta E_{elstat}^{ref}$	$\Delta E_{exch}^{ref}$	$\Delta E_{non-disp}^{C-CCSD}$	$\Delta E_{disp}^{C-CCSD}$	$\Delta E_{int}^{C-\left(T\right)}$
Gemcitabine–Adenine	−17.68	0.92	76.44	−81.51	−12.62	5.45	−5.33	−0.93
Gemcitabine–Guanine	−27.59	2.00	109.34	−119.48	−17.44	7.53	−6.50	−1.03
Gemcitabine–Cytosine	−22.59	1.57	93.33	−101.03	−14.89	6.75	−5.38	−0.82
Gemcitabine–Thymine	−19.96	1.44	79.74	−86.60	−13.11	6.60	−5.40	−0.81
Gemcitabine–Uracil	−20.65	1.27	82.13	−89.41	−13.36	6.63	−5.29	−0.81

**Table 6:** NBO analysis of the drug gemcitabine–nucleobase complexes and the hydrogen bond energy (E_HB_) computed using QTAIM theory.

Donor NBO (i)	Acceptor NBO (j)	E(2) kcal mol^−1^	F(I,j) a.u.	E_HB_
GEM				
LP(1) O29	BD*(1) O22-H23	3.03	0.06	5.46
LP(1) O29	BD*(1) C15-H16	0.53	0.02	3.10
Gemcitabine–Adenine complex				
LP(1) O29	BD*(1) O22-H23	2.92	0.06	5.54
LP(1) O29	BD*(1) C15-H16	0.50	0.22	2.97
Gemcitabine–Guanine complex				
LP(1) O29	RY*(1) H23	0.84	0.04	5.39
LP(1) O29	RY*(1) H23	0.60	0.04	-
LP(1) O29	BD*(1) C15-H16	-	-	2.98
Gemcitabine–Cytosine complex				
LP(1) O29	BD*(1) O22-H23	3.21	0.06	5.92
LP(1) O29	BD*(1) C15-H16	-	-	3.01
Gemcitabine–Thymine complex				
LP(1) O29	BD*(1) O22-H23	2.69	0.05	5.39
LP(1) O29	BD*(1) C15-H16	-	-	2.74
Gemcitabine–Uracil complex				
BD*(2) C28-29	BD*(1) O22-H23	0.69	0.04	5.62
LP(1) O29	BD*(1) C15-H16	-	-	2.90

## Data Availability

Data are contained within the article and Supplementary Materials.

## Supplementary Materials

The following supporting information can be downloaded at https://www.mdpi.com/article/10.3390/molecules30132732/s1: Figures S1–S5: Optimized geometries of the low-lying isomers of gemcitabine–adenine, gemcitabine–guanine, gemcitabine–cytosine, gemcitabine–thymine. and gemcitabine–uracil; Figure S6: HOMO and LUMO orbitals of the nucleobase, the gemcitabine drug, and the most stable gemcitabine–nucleobase complexes; Figure S7: NCI and RDG plots of the nucleobases and gemcitabine.

## Abbreviations

The following abbreviations are used in this manuscript:

CCSD(T)	Coupled Cluster Singles Doubles with perturbative Triples
DFT	Density Functional Theory
DLPNO	Domain-Based Local Pair Natural Orbital
HOMA	Harmonic Oscillator Measure of Aromaticity
NICS	Nucleus-Independent Chemical Shift
NCI–RDG	Non-Covalent Interaction–Reduced Density Gradient
QTAIM	Quantum Theory of Atoms in Molecules
