# Supplementary material for: Intramolecular Versus Intermolecular Bonding in Drug Gemcitabine and Nucleobases: A Computational Study
Source: Molecules. 2025 Jun 25;30(13):2732. doi: 10.3390/molecules30132732 (PMC12251269; doi:10.3390/molecules30132732)
Supplement: Supplementary file 1 [file molecules-30-02732-s001.zip › molecules-3701508-supplementary.pdf]

# Supporting information

## Intramolecular Versus Intermolecular Bonding in Drug Gemcitabine and Nucleobases: A Computational Study

**Natarajan Sathiyamoorthy Venkataramanan [1,*], Ambigapathy Suvitha [2] and Ryoji Sahara [3,*]**

[1] Department of Chemistry, School of Engineering, Dayanada Sagar University, Bangalore 562 112, India
[2] Department of Chemistry, SKP Engineering College, Tiruvannamalai 606 601, India; suvithaa@gmail.com
[3] Computational Structural Materials Group, Research Center for Structural Materials, National Institute for Materials Science (NIMS), 1-2-1, Sengen, Tsukuba 305-0047, Japan
* Correspondence: venkataramanan-chem@dsu.edu.in or nsvenkataramanan@gmail.com (N.S.V.); sahara.ryoji@nims.go.jp (R.S.)

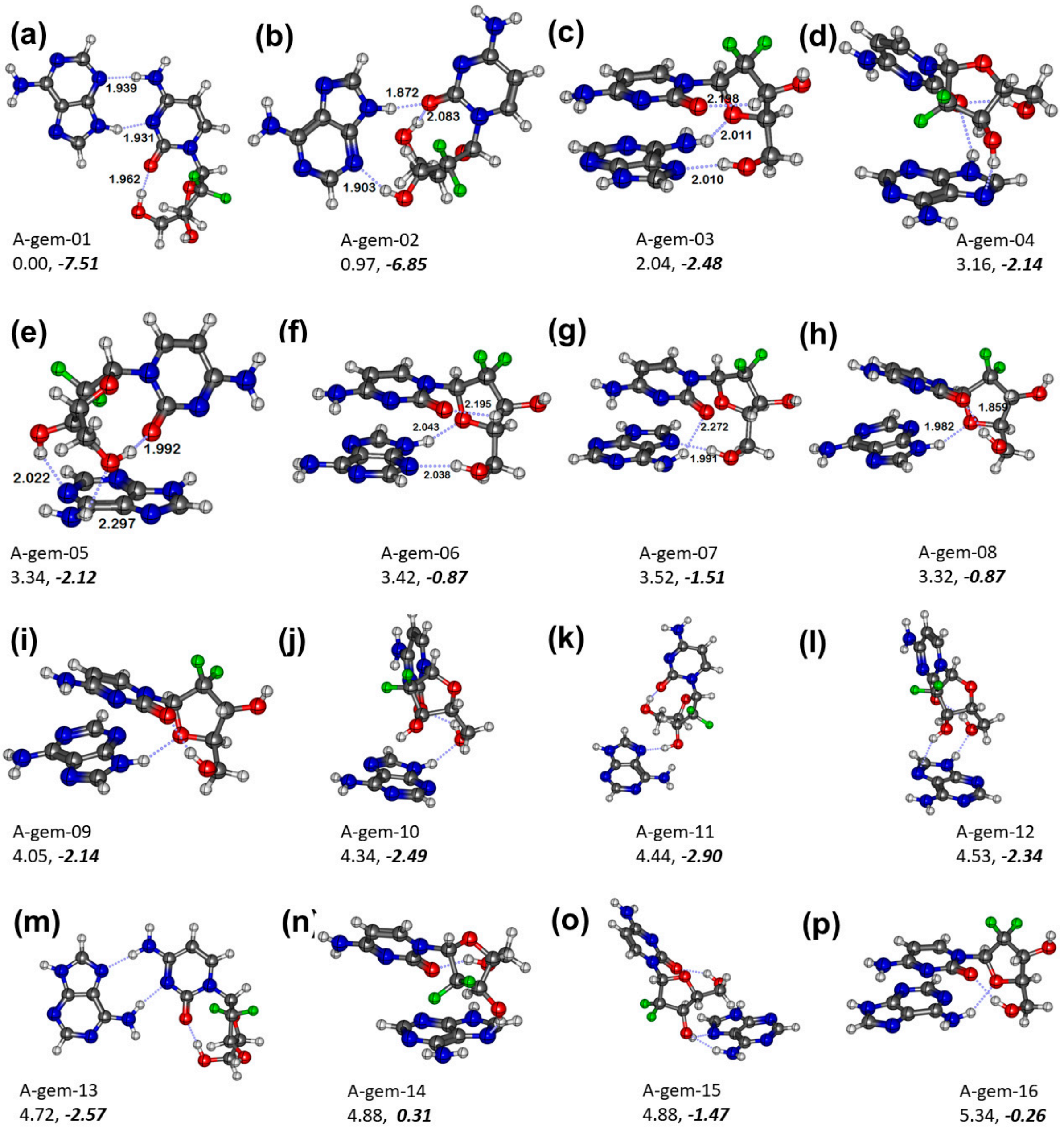


Figure S1. Optimized geometries of the low-lying isomers (a-p) for the gemcitabine-Adenine complexes computed using M06-2X functional. The relative energy of the isomer and its Gibbs free energy (in italics) in kJ/mol are provided in the insert.

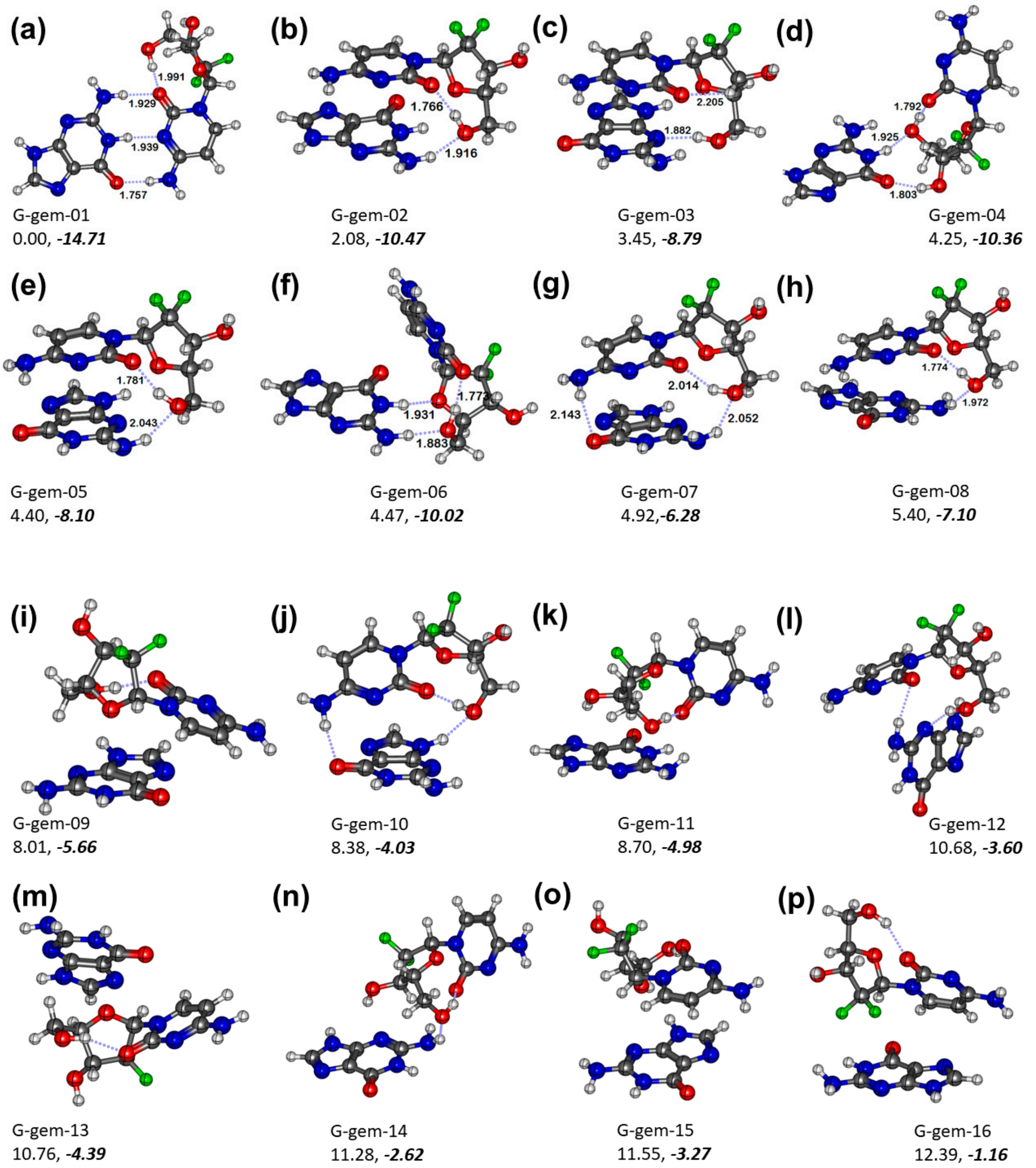


Figure S2. Optimized geometries of the low-lying isomers (a-p) for the gemcitabine-Guanine complexes computed using M06-2X functional. The relative energy of the isomer and its Gibbs free energy (in italics) in kJ/mol are provided in the insert.

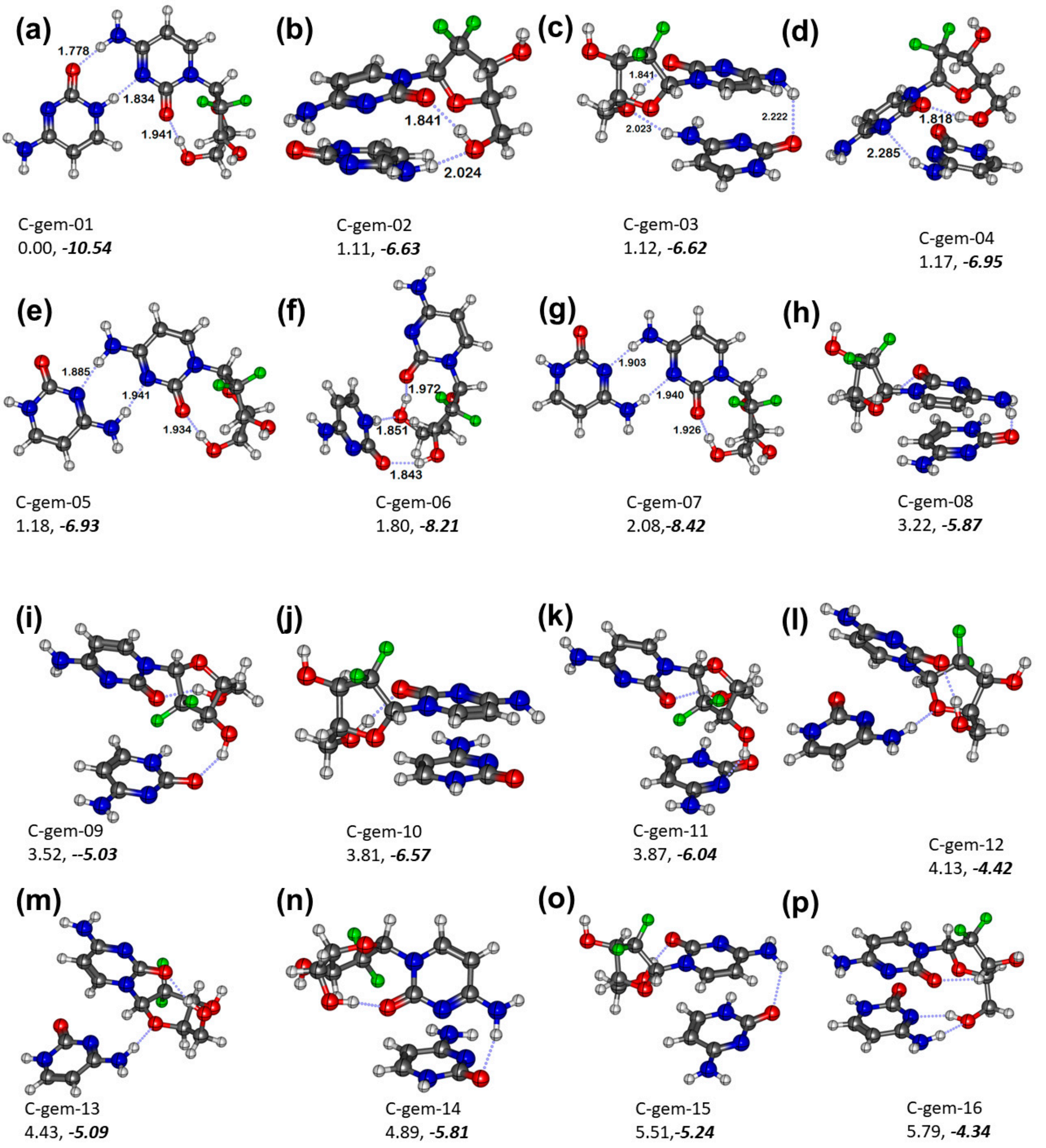


Figure S3. Optimized geometries of the low-lying isomers (a-p) for the gemcitabine-cytosine complexes computed using M06-2X functional. The relative energy of the isomer and its Gibbs free energy (in italics) in kJ/mol are provided in the insert.

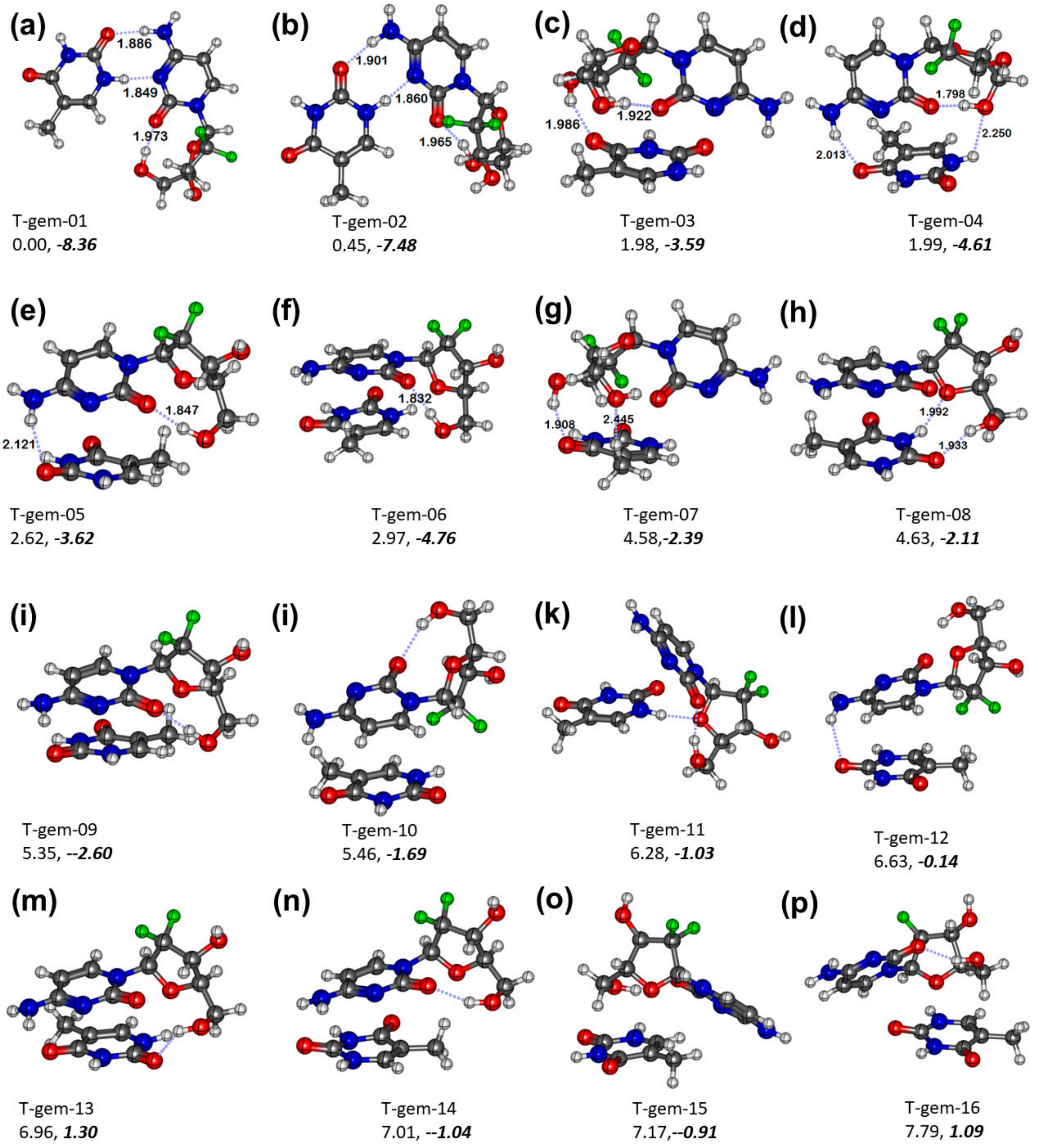


Figure S4. Optimized geometries of the low-lying isomers (a-p) for the gemcitabine-Thymine complexes computed using M06-2X functional. The relative energy of the isomer and its Gibbs free energy (in italics) in kJ/mol are provided in the insert.

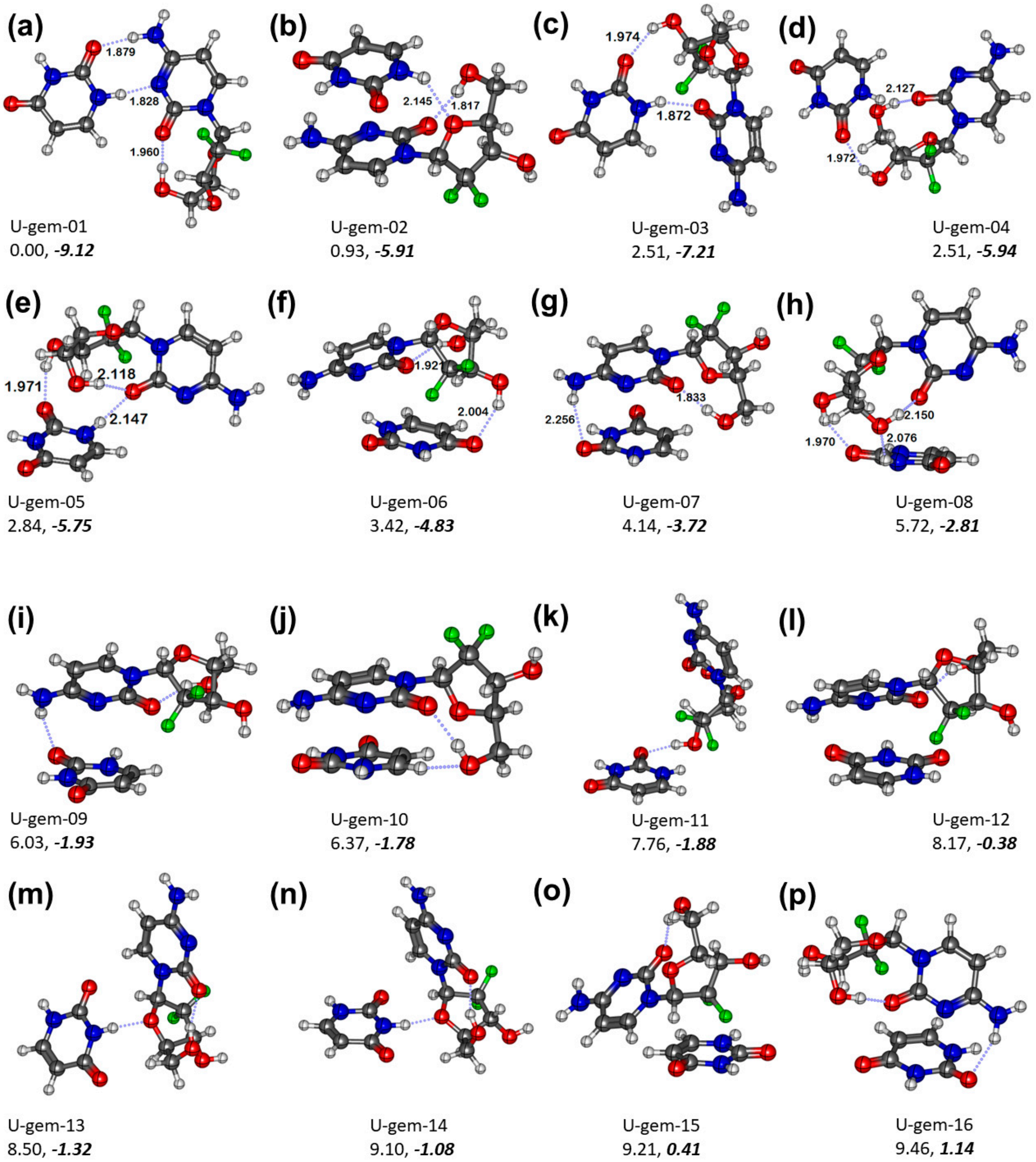


Figure S5. Optimized geometries of the low-lying isomers (a-p) for the gemcitabine-Uracil complexes computed using M06-2X functional. The relative energy of the isomer and its Gibbs free energy (in italics) in kJ/mol are provided in the insert.

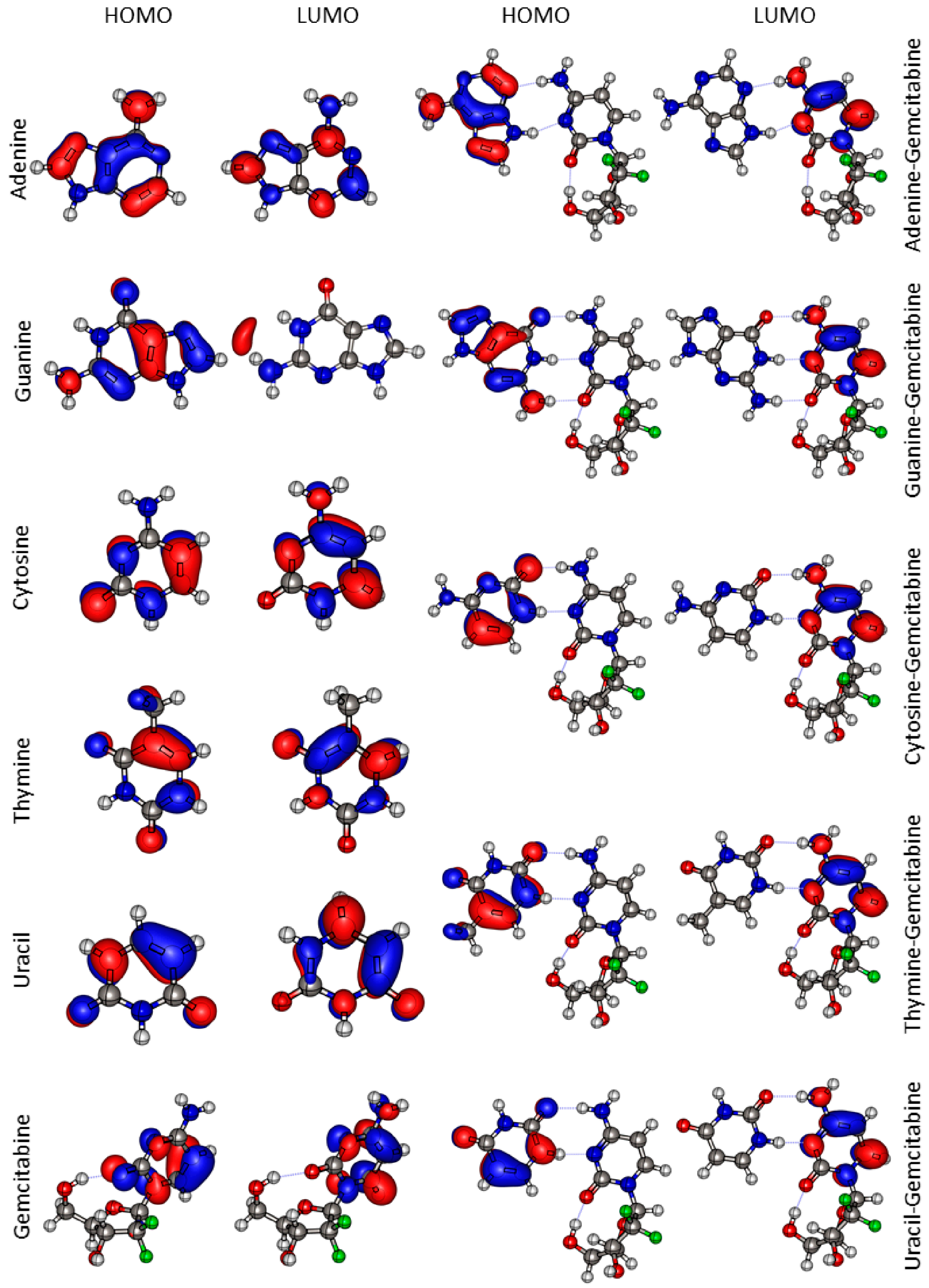


Figure S6. HOMO and LUMO orbitals of nucleobase, gemcitabine drug, and the most stable gemcitabine-nucleobase complexes

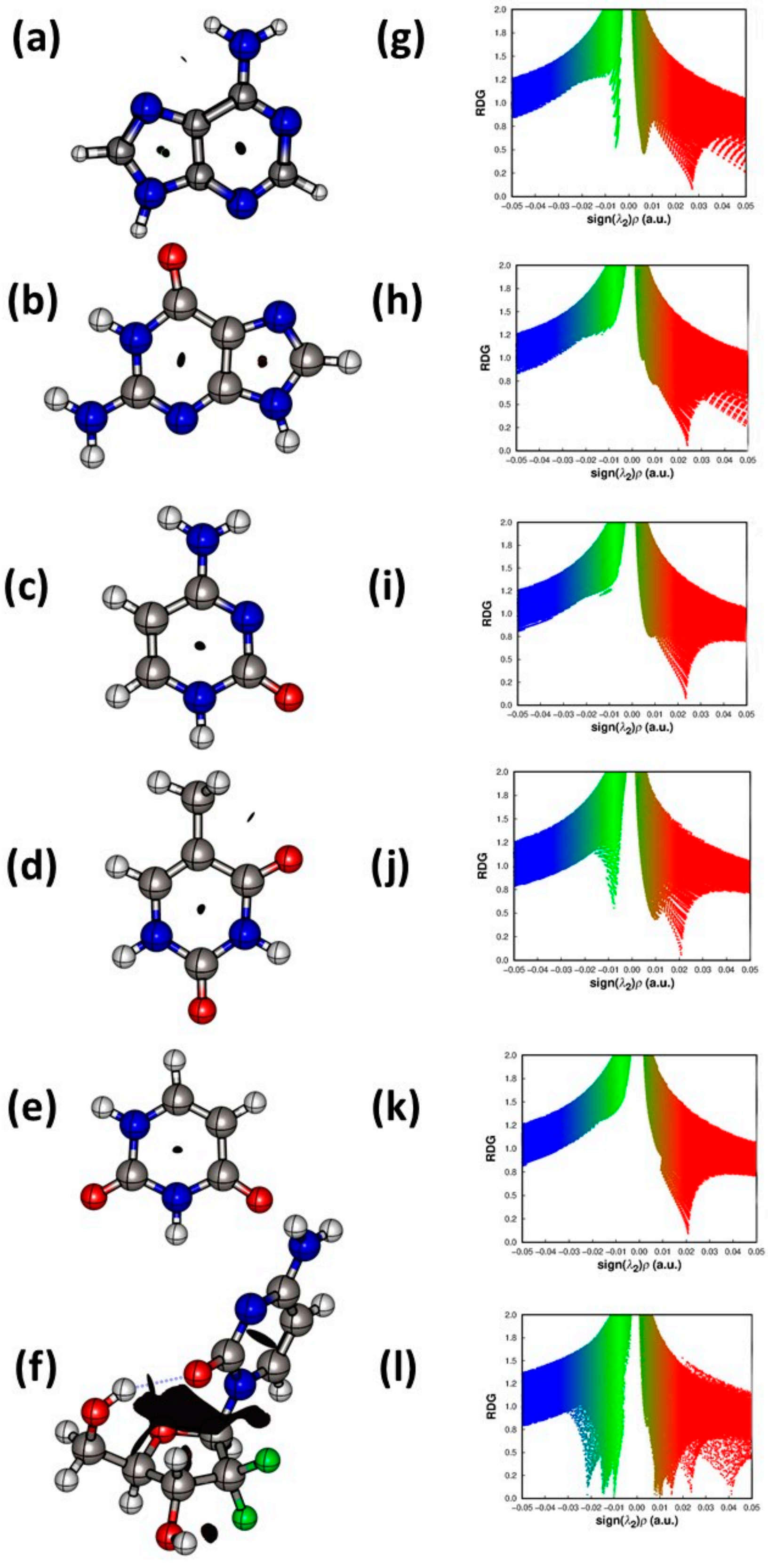


Figure S7. NCI isosurface of (a-e) nucleobases, (f) gemcitabine drug, (g-k) RDG plot for nucleobases and (l) RDG plot for gemcitabine drug.
